# Evaluation of the Delivery of a Live Attenuated Porcine Reproductive and Respiratory Syndrome Virus as a Unit Solid Dose Injectable Vaccine

**DOI:** 10.3390/vaccines10111836

**Published:** 2022-10-30

**Authors:** Ellie Hayhurst, Emily Rose, Miriam Pedrera, Jane C. Edwards, Natalia Kotynska, Daisy Grainger, Yashar Sadigh, John Flannery, Ludo Bonnet, Ritwik Ritwik, Pawan Dulal, M. Keith Howard, Simon P. Graham

**Affiliations:** 1 The Pirbright Institute, Pirbright GU24 0NF, UK; 2 Enesi Pharma, Abingdon OX14 4SA, UK

**Keywords:** porcine reproductive and respiratory syndrome virus, live attenuated vaccine, solid dose vaccine formulation, immunogenicity, pig

## Abstract

Solid dose vaccine formulation and delivery systems offer potential advantages over traditional liquid vaccine formulations. In addition to enhanced thermostability, needle-free delivery of unit solid dose injectable (USDI) vaccines offers safe, rapid, and error-free administration, with applicability to both human and animal health. Solid dose formulation technologies can be adapted for delivery of different vaccine formats including live attenuated vaccines, which remain the ‘gold standard’ for many disease targets. Porcine reproductive and respiratory syndrome viruses (PRRSV) cause one of the most economically important diseases affecting the global pig industry. Despite several shortcomings, live attenuated vaccines are widely used to control PRRSV. We optimised a freeze-dried USDI formulation of live attenuated PRRSV-1, which fully retained infectious titre, and evaluated its immunogenicity in comparison to virus delivered in liquid suspension via intramuscular and subcutaneous needle inoculation. Pigs vaccinated with the USDI formulation displayed vaccine viraemia, and PRRSV-specific antibody and T cell responses comparable to animals immunised with the liquid vaccine. The USDI vaccine formulation was stable for at least 6 months when stored refrigerated. These data demonstrate the potential for a solid dose vaccine delivery system as an alternative to conventional needle-syringe delivery of live attenuated PRRSV vaccines.

## 1. Introduction

The COVID-19 pandemic has highlighted the need for thermostable vaccines that are quick, simple, and safe to administer. This is broadly true for both human and animal vaccines. A number of technologies have emerged or are under development to meet this challenge and replace traditional needle-inoculated liquid dose vaccines [1]. These include microneedle patches for transcutaneous immunisation [2,3,4]; dry powders for intranasal immunisation [5,6,7] and tableted solid doses for oral or subcutaneous immunisation [8,9,10]. Unit solid dose vaccine delivery is advantageous over liquid-needle delivery as it potentially reduces the requirement for cold-chain storage, extends the shelf life of vaccines and provides enhanced immunogenicity and dose-sparing.

Porcine reproductive and respiratory virus (PRRSV) 1 and 2 (Betaarterivirus suid 1 and 2) are two species of enveloped, single stranded, positive-sense RNA viruses within the Betaarterivirus genus of the *Arteriviridae* family [11]. Clinical symptoms following infection with PRRSV included reproductive failure, growth reduction, post-weaning pneumonia and increased mortality due to respiratory disease [12]. PRRS remains one of the most economically important diseases affecting the global swine industry and is estimated to cost the USA alone around USD 560 million per year [13]. Due to a high mutation rate, there is considerable genetic/antigenic diversity within both PRRSV-1 and PRRSV-2, which poses a significant challenge to the control of the virus through vaccination. Both inactivated and live attenuated PRRSV vaccines are commercially available. Live attenuated vaccines are more widely used due to enhanced immunogenicity, which provides efficacy against related strains but variable protection against divergent strains [14]. Live attenuated PRRSV are typically formulated as freeze-dried powders to improve storage and are reconstituted in diluent immediately prior to inoculation by intramuscular needle injection or intradermal needle-free liquid jet delivery device [15,16].

Enesi Pharma has developed the unit solid dose injectable (USDI) vaccine technology, which includes a device containing a multi-use actuator and a single-use unit dose cassette, which contains a precision engineered unit solid dose vaccine. Pushing the device against the skin activates the actuator, automatically delivering the fully dissolvable USDI into the subcutaneous layer of the skin. The USDI vaccine technology has previously demonstrated enhanced immune response compared to standard injection in small animal models immunised with inactivated and subunit protein-based vaccines [17]. Delivery of live attenuated vaccines to large animals as USDI is being reported here for the first time as a proof of principle. The aim of this study, in which a needle and trocar was used in place of a multi-use actuator to deliver a prototype USDI, was to establish a USDI formulation of live attenuated PRRSV which retains infectious titre and provides comparable immunogenicity to a standard delivery of virus in liquid suspension by needle-syringe inoculation.

## 2. Materials and Methods

### 2.1. Cell Culture

African green monkey kidney derived MARC-145 cells were cultured in 75 cm^2^ tissue culture flasks and maintained in Dulbecco’s Modified Eagle’s Medium (DMEM) supplemented with 10% foetal bovine serum (FBS) and 1% penicillin-streptomycin-gentamycin (cDMEM) (all Thermo Fisher Scientific, Loughborough, UK) in a humidified incubator at 37 °C and 5% CO_2_. Cells were passaged 2–3 times weekly depending on the confluency. cDMEM was aspirated and the cell monolayer washed once with phosphate buffered saline (PBS). Cells were then detached using 4 mL of 0.05% trypsin-EDTA (Sigma-Aldrich, Merck, Poole, UK) for 5 min at 37 °C. Trypsin-EDTA was neutralised with 6 mL of cDMEM. Cell density was calculated using a haemocytometer and 1 × 10^6^ cells transferred to a new 75 cm^2^ tissue culture flask for continued culture.

### 2.2. PRRSV-1 Propagation and Titration

The MARC-145 cell attenuated Western European PRRSV-1 subtype 1 Olot/91 strain [18] (kindly provided by Dr Jean-Pierre Frossard, Virology Department, Animal and Plant Health Agency, Weybridge, UK) was used as a model live attenuated PRRSV vaccine. PRRSV-1 Olot/91 was propagated in MARC-145 cells by inoculating confluent 850 cm^2^ roller bottle cultures (Thermo Fisher Scientific) at a multiplicity of infection (MOI) of 0.1 and culturing for 4–5 days at 37 °C in DMEM supplemented with 1% FBS and 1% penicillin-streptomycin (propagation media). To harvest the virus, supernatant was removed, and the cells lysed in residual volume by freeze-thaw at −20 °C. The lysed cells were pooled with the culture supernatant and clarified by centrifugation at 4700 rpm (TX-750 rotor, Beckman Coulter, High Wycombe, UK) for 15 min at 4 °C, aliquoted and stored at −80 °C. Mock virus supernatants were prepared in the same way but with inoculation of roller bottle cultures with medium containing no virus. 

To titrate the virus, 5- or 10-fold serial dilutions were prepared in cDMEM and added to 96 well flat-bottom plates (Nunc, Thermo Fisher Scientific) containing 1.5 × 10^4^ MARC-145 cells/well and incubated for 3 days at 37 ^o^C and 5% CO_2_. MARC-145 cell monolayers were washed with PBS and fixed using 2% paraformaldehyde in PBS with 1% Triton X-100 (all Sigma-Aldrich) for 10 min at room temperature. Cells were washed three times with PBS containing 0.2% Tween-20 (PBSt) and incubated with 10% goat serum (Thermo Fisher Scientific) diluted in PBSt for 30 min at room temperature. This blocking solution was removed and replaced with anti-PRRSV nucleocapsid (N) protein mouse monoclonal antibody (mAb) SDOW-17 (RTI, Brookings, USA) diluted 1:4000 in PBSt and incubated for 60 min at room temperature. Following three washes in PBSt, polyclonal goat anti-mouse immunoglobulin-horseradish peroxidase (HRP) conjugate (Thermo Fisher Scientific), diluted 1:4000 in PBSt, was added and incubated at room temperature for 60 min. After washing three times in PBSt, AEC (3-amino-9-ethylcarbazole) prepared in AEC substrate buffer (Sigma-Aldrich) was added, and the cells were incubated for 20 min at room temperature. Finally, the cells were washed in PBS and examined under a light microscope to discern PRRSV-infected cells by the presence of orange cytosolic staining. 

### 2.3. Formulation Development

A variety of sugar and sugar-alcohol excipients including sucrose, trehalose, sorbitol, mannitol, in combination with polymeric excipients such as gelatin hydrolysate, recombinant human serum albumin, polyethylene glycol (PEG), and polyvinyl pyrrolidone (PVP) (Fisher Scientific, UK) were initially screened to produce optimum freeze-dried matrix (FDM) in combination with the virus propagation medium. Five different excipient compositions of the PRRSV-1 vaccine were prepared with a fixed combination of stabilising excipients gelatin, albumin and sorbitol and varying combination of mannitol, sucrose, trehalose, PEG and PVP (Table 1) prior to freeze drying. Solid doses were manufactured using three different manufacturing conditions which included cold extrusion (process 1) and micro-tableting with compaction force of 10 kg (process 2), or 100 kg (process 3) in the presence of bulking excipients such as dextran, carboxymethyl cellulose, and magnesium stearate to provide strength as previously described [19,20]. The retention of infectivity of the vaccine in FDMs and solid doses was assessed by TCID_50_ to enable selection of the final formulation for the in vivo study. As the retention of infectivity of the vaccine on all five FDMs was within the variability of the assay, formulation 4 was selected for solid dose manufacturing due to its simpler and more cost-effective composition. Upon selection, three rounds of manufacture and TCID_50_ analysis were conducted to confirm titre and reproducibility of the initial solid dose.

Samples for the stability study were stored at 2–8 °C (uncontrolled humidity), 25 °C (60% relative humidity; RH), 40 °C (75% RH) inside nitrogen purged foil pouches (Milfoil, Moore & Buckle, St. Helens, UK).

### 2.4. Evaluation of the Infectious Titres of Freeze-Dried and Solid Dose PRRSV-1

Freeze dried matrix powders and solid dose formulations of PRRSV-1 Olot/91were reconstituted by addition of serum-free DMEM and incubated at room temperature for 30 min to allow samples to dissolve. Sample volumes were then adjusted to a predicted titre of 10^5^ TCID_50_/mL, serially diluted 5-fold, and titrations performed using MARC-145 cells in 96 well plates as described above.

### 2.5. Immunisation of Pigs with Solid and Liquid Doses of PRRSV-1 Olot/91

Animal work was approved by the Animal and Plant Health Agency and The Pirbright Institute Animal Welfare and Ethics Committees and conducted in accordance with the UK Animals (Scientific Procedures) Act 1986 (Project Licence P6F09D691). Eighteen, PRRSV naïve, female, 8–10-week-old pigs were randomly assigned to three test groups (n = 6) receiving either a liquid subcutaneous (s.c.) dose, a liquid intramuscular dose (i.m.) or a USDI (s.c.) of PRRSV-1 Olot/91. Solid doses were prepared at 1 × 10^5^ TCID_50_/dose. A liquid suspension of PRRSV-1 Olot/91 was diluted to 1 × 10^5^ TCID_50_/mL in PBS from a virus stock at 1 × 10^7^ TCID_50_ and delivered 1 mL/dose). USDI were inserted into the subcutaneous layer of the skin using a needle-trocar device (RFID microchip Mini, Chiphandel, Germany). Liquid inocula were prepared in luer-lock syringes with 19 G 1” needles. All pigs were first sedated by i.m. injection with 2 mg/kg Stresnil™ (azaperone, Elanco) and inoculation sites treated with a topical local anaesthetic (Emla cream). i.m. inoculations were administered to the brachiocephalic muscle and s.c. inoculations under the skin behind the ear. Animals were clinically scored [21], and rectal temperatures measured daily until 13 days post-immunisation (DPI). Blood samples were collected into BD SST and heparin vacutainers (Thermo Fisher Scientific) from animals on 0, 3, 7, 10, 14, 17, 21, 28, and 35 DPI to assess viremia and immune responses. 

### 2.6. Isolation of Serum and Peripheral Blood Mononuclear Cells

Serum was prepared by centrifugation of BD SST vacutainers at 1300× *g* for 10 min at room temperature and stored at −80 °C. Peripheral blood mononuclear cells (PBMCs) were isolated by diluting heparinised blood 1:1 in PBS and layering over Histopaque 1.077 using Leucosep blood separation tubes (both Merck). Tubes were centrifuged at 800 × *g* for 15 min with no brake. PBMC were harvested and washed with PBS by centrifugation at 300× *g* for 10 min. The supernatant was removed, and the remaining cell pellet was resuspended in 5 mL of RBC Lysis Buffer (BioLegend, London, UK) and incubated for 5 min at room temperature. The PBMCs were then washed twice with PBS and centrifugation at 500× *g* for 5 min. The cell pellet was resuspended in RPMI-1640 medium containing 10% FBS, 1% penicillin-streptomycin-gentamycin and 0.1% 2-mercaptoethanol (50 mM) (cRPMI) (all Thermo Fisher Scientific). Cell density was calculated by diluting an aliquot of cells fixed in 4% paraformaldehyde 1:20 in PBS and running through a volumetric flow cytometer (MACSQuant Analyzer, Miltenyi Biotec, Bisley, UK). 

### 2.7. Assessment of PRRSV-1 Viraemia by Quantitative RT-PCR

RNA was isolated from serum using the MagMAX™ CORE Nucleic Acid Purification Kit and KingFisher Flex automated extraction instrument (both Thermo Fisher Scientific). PRRSV RNA measured by reverse transcription quantitative PCR (RT-qPCR; VetMAX™ PRRSV EU & NA 2.0 RT-PCR kit, Thermo Fisher Scientific). The level of viraemia was expressed as the reduction in the number of PCR cycles beyond the set-negative level of 40-cycles that were required to exceed the threshold fluorescence value.

### 2.8. IFN-γ ELISpot Assay

ELISpot plates (MAIPS4510, Merck Millipore, Gillingham, UK) were activated by addition of 35% ethanol for 1 min at room temperature. After washing thrice with PBS, anti-porcine IFN-γ mAb (clone P2G10, BD Pharmingen, BD Biosciences, Oxford, UK) diluted 1:500 in PBS was added and incubated overnight at 4 °C. The excess coating antibody was removed by washing three times in PBS. cRPMI was added to block the plates by incubation for at least 1 h at 37 °C, 5% CO_2_. PBMCs were then added at a density of 5 × 10^5^ cells/well in cRPMI. Cells were stimulated with PRRSV-1 Olot/91 at an MOI of 0.1, an equivalent volume of mock virus supernatant, or 10 µg/mL concanavalin A (Sigma-Aldrich) and incubated for 18 h at 37 °C, 5% CO_2_. Cells were lysed by addition of cold MilliQ water for 5 min at 4 °C. This was followed by washing three times in PBS with 0.05% Tween-20 (PBSte), before the addition of biotinylated anti-porcine IFN-γ mAb (clone P2C11, BD Pharmingen) diluted 1:3000 in PBSte with 1% FBS and incubation at 37 °C, 5% CO_2_ for 90 min. After washing five times in PBSte, streptavidin-alkaline phosphatase (R&D Systems, Bio-Techne, Abingdon, UK), diluted 1:60 in PBSte with 1% FBS, was added and incubated for a further 90 min at room temperature in the dark. Plates were washed three times with PBSte and three times using PBS, before addition of BCIP/NBT substrate (R&D Systems) and incubation at room temperature for 30 min. The reaction was stopped using MilliQ water. The plastic base was then removed and well membranes washed with copious amounts of water. The plates were air-dried, and then read and analysed using the AID ELISpot System Classic Reader (AID, Strassberg, Germany).

### 2.9. Intracellular Cytokine Staining Assay

PBMC in cRPMI were seeded at 1 × 10^6^ cells/well in 96-well round bottom plates (Nunc, Thermo Fisher Scientific) and stimulated with PRRSV-1 Olot/91 at a MOI = 0.1, in triplicate wells. PBMC incubated in triplicate wells with an equivalent volume of mock virus supernatant served as a negative control. After 18 h at 37 °C, 5% CO_2_, BD GolgiPlug (1:1000; BD Biosciences) was added and cells further incubated for 6 h at 37 °C. PBMC were then surface stained with Zombie NIR fixable viability stain (BioLegend), CD3-FITC mAb (clone BB23-8E6-8C8, BD Biosciences), CD4-PerCP-Cy5.5 mAb (clone 74-12-4, BD Bioscience) and CD8α-PE mAb (clone 76-2-11, BD Bioscience), and intracytoplasmically stained with IFN-γ-Alexa Fluor 647 mAb (clone CC302, Bio-Rad Antibodies, Kidlington, UK) and TNF-α-Brilliant Violet 421 mAb (clone Mab11, BioLegend) as described previously [22]. Cells were analysed using a MACSQuant Analyzer 10 flow cytometer (Miltenyi Biotec).

### 2.10. PRRSV-1 N-Protein ELISA

A commercial diagnostic ELISA kit, PrioCHECK^®^ PRRSV Ab porcine ELISA (Thermo Fisher Scientific), was used to detect antibodies against PRRSV-1 in serum, as described by the manufacturer. In brief, serum, and positive/negative control samples, diluted 1:20 in sample diluent, were incubated in the ELISA plate for 60 min at room temperature before washing four times in wash fluid. Provided conjugate solution was then diluted 1:30 in conjugate diluent and added. Following incubation for 60 min at room temperature, the test plate was washed four times using wash fluid. The chromogenic 3,3’,5,5’-tetramethylbenzidine (TMB) substrate was then added and incubated for 20 min at room temperature. Stop solution was added and absorbance measured at 450 nm and 620 nm using a spectrophotometer (GloMax®-Multi Detection System, Promega, Southampton, UK).

### 2.11. PRRSV Neutralization Assay

Serum was heat inactivated by incubation at 56 ^o^C for 30 min and clarified by centrifugation in a minifuge at 13,000 rpm for 10 min. Serum was serially diluted 2-fold in cDMEM and mixed with an equal volume of cDMEM (serum only) or cDMEM containing 400 TCID_50_ of PRRSV-1 Olot/91 (serum + virus) in 96-well flat bottom plates. Plates were incubated at 37 °C, 5% CO_2_ for 1 h. MARC-145 cells were then added at a density of 1.5 × 10^4^ cells/well. An additional plate was prepared to function as a control with only virus being added in a 10-fold dilution series beginning with 400 TCID_50_/well and ending with no virus. Each plate was then incubated 37 °C, 5% CO_2_ for 48 h. MARC-145 cells were then subjected to immunoperoxidase staining as described above with the exception that TMB substrate (Sigma-Aldrich) was added, and the cells were incubated for 5–10 min at room temperature in the dark. 1M sulfuric acid was added to stop the reaction and absorbance was measured at 600 nm and 450 nm using a spectrophotometer (GloMax®-Multi Detection System, Promega). Percent neutralisation was calculated for each serum dilution using the formula: 100 − [(OD serum + virus − OD serum only)/(OD virus only − OD cells only) × 100]. Data were interpolated to determine the reciprocal serum dilution that neutralised virus infection by 50% (50% neutralising titre). 

### 2.12. Data Analysis

Date were graphically and statistically analysed using GraphPad Prism 8.2.1 (GraphPad Software, Inc., La Jolla, CA, USA). Flow cytometry and ELISpot data were analysed using FlowJo 10.7.1 software (BD Biosciences) and ELISpot iSpot 5 software (AID), respectively. One- or two-way ANOVA or a mixed-effects model were used to analyse datasets. Virus and antibody titre data were log_10_ transformed before analysis. A *p* value < 0.05 was considered statistically significant.

## 3. Results

### 3.1. In Vitro Analysis of Lyophilised and Solid Dose Formulations of PRRSV MLV

**Figure 1:**
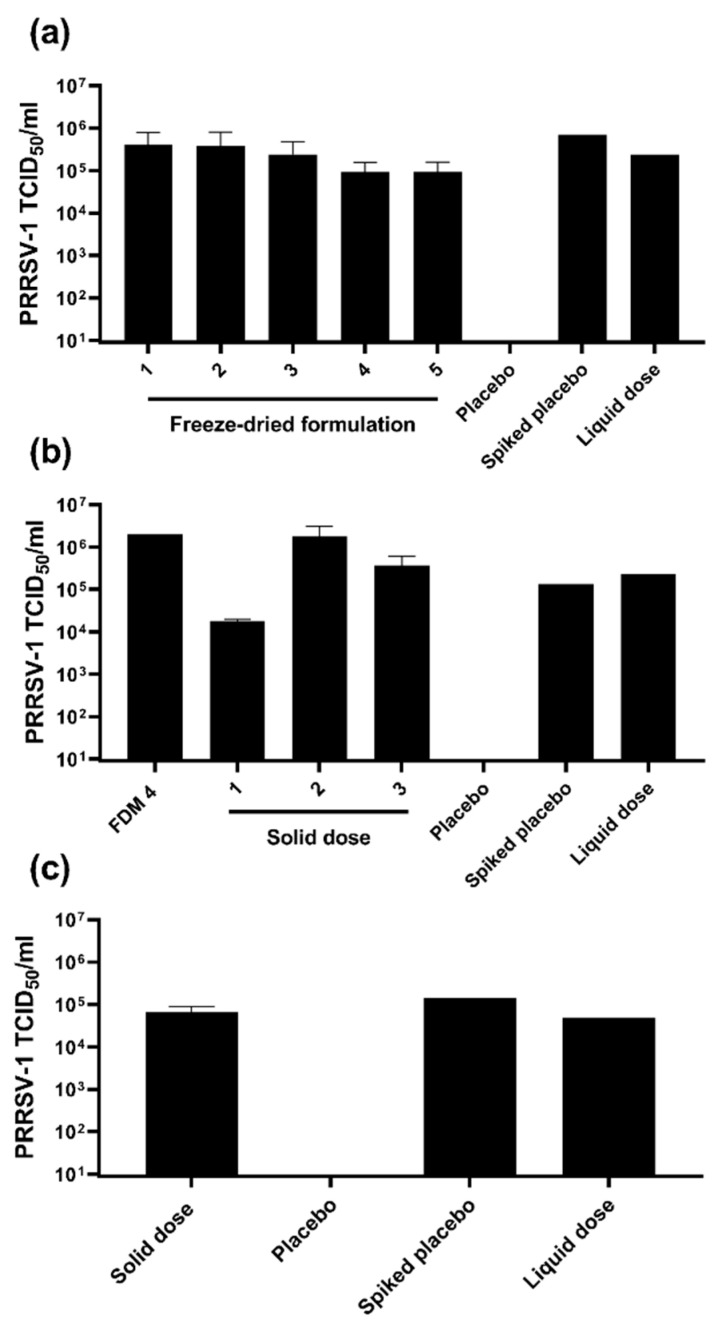
Assessment of the retention of infectious titre in freeze dried matrix (FDM) powder and solid dose formulations of live attenuated PRRSV-1. (**a**) Duplicate samples of five different FDM formulations (1–5) of PRRSV-1 Olot/91 were reconstituted in DMEM, adjusted to a predicted titre of 1 × 10^5^ TCID_50_/mL, titrated on MARC-145 cells and titres determined following immunoperoxidase staining. A placebo FDM served as a negative control. A reconstituted placebo FDM spiked with PRRSV-1 and untreated PRRSV-1 (liquid dose) served as positive controls. (**b**) PRRSV-1 infectious titres were assessed in duplicate (solid dose 1), or quadruplicate (solid doses 2 and 3) reconstituted samples of solid doses manufactured from FMD 4 using three different manufacturing processes (solid dose 1–3). (**c**) The infectious titres in duplicate reconstituted solid doses manufactured (by process 3) for subsequent in vivo testing were determined.

PRRSV-1 Olot/91 was propagated in roller bottle cultures in medium with low (1%) FBS to a titre of ~10^7^ TCID_50_/mL, aliquoted and stored at −80 °C. Five different FDM powder formulations of this virus batch were initially manufactured and assessed for retention of infectious titre. Following reconstitution in DMEM, virus titres were determined using MARC-145 cells (Figure 1a). All five freeze-dried matrix formulations retained a titre comparable to positive control aliquots of the same virus batch stored at −80 °C within the variability of the assay of approximately 0.7 log_10_ TCID_50_/mL. Based on the retention of infectivity of the virus and better compatibility of excipients for solid dose manufacturing, FDM4 was selected and USDI were manufactured using three different processes (Figure 1b). Virus titres were maintained in solid doses manufactured by all three processes, however process 1 (solid dose 1) displayed significantly lower titres compared to the other two other processes (*p* < 0.05). Manufacturing process 3 was selected to produce a solid dose formulation for subsequent in vivo testing for the superior mechanical strength (measured by Shimadzu AGS-X combined with Trapezium X Software) required for use as USDI, compared to those manufactured by Process 2. Evaluation of these solid doses confirmed they retained the expected infectious titre (Figure 1c).

### 3.2. Analysis of the Immunogenicity of a Unit Solid Dose Injectable Vaccine Formulation of PRRSV MLV

**Figure 2:**
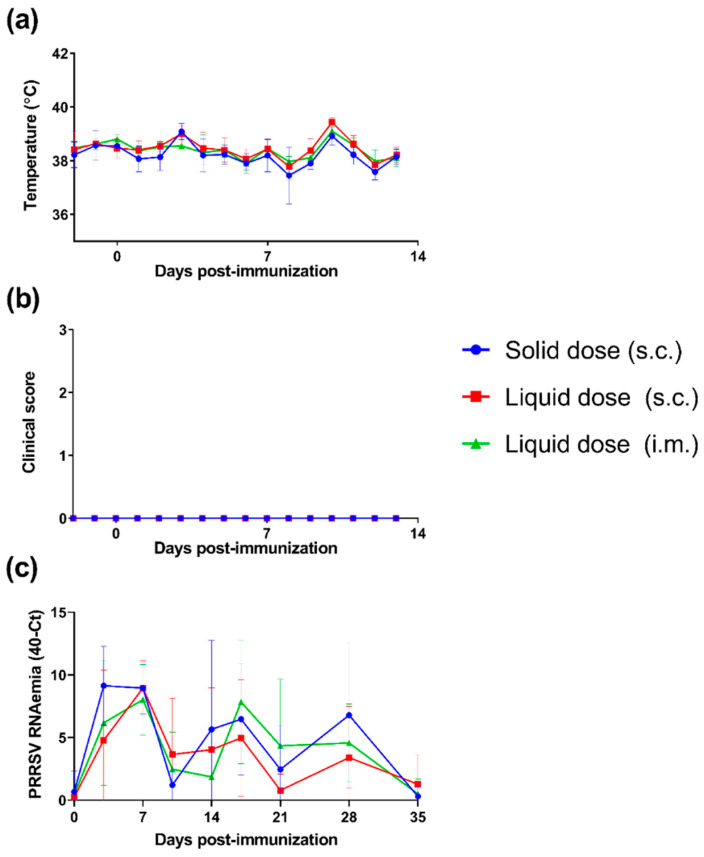
Assessment of clinical signs and viraemia following immunisation of pigs with a unit solid dose injectable formulation of live attenuated PRRSV-1. Three groups of pigs (n = 6) were immunised by s.c. inoculation of a USDI formulation of 10^5^ TCID_50_ of PRRSV-1 Olot/91, or by s.c. or i.m. inoculation of a comparable dose of PRRSV-1 Olot/91 (s.c. liquid dose and i.m. liquid dose, respectively). (**a**) Rectal temperatures and (**b**) clinical signs were scored daily from -2 to 13 days post-immunisation. (**c**) Levels of PRRSV-1 RNA circulating in blood were assessed by RT-qPCR analysis of longitudinally collected sera. RT-qPCR data are presented as 40—cycle threshold (Ct). Mean data ± SD are shown for each vaccine group.

Following demonstration that the USDI formulation of PRRSV-1 Olot/91 successfully retained the expected infectious titre, an in vivo study involving three groups of 6 pigs was conducted to compare the immunogenicity of the USDI formulation with a comparable standard dose of virus suspended in liquid medium. One group received the USDI formulation, delivered s.c. using a needle trocar, while a second group received the control s.c. injection of a liquid suspension of PRRSV-1 Olot/91. The final group received an i.m. injection of liquid PRRSV-1 Olot/91, since it was unclear whether delivery of virus via the s.c. route would generate the same immune response as the established i.m. delivery method. Following vaccination, pigs were clinically monitored, and blood samples were collected longitudinally to assess the immune responses and vaccine virus viraemia. Following vaccination with either vaccine formulation or route, pigs showed similar fluctuations in daily rectal temperatures which did not deviate significantly from the normal body temperature range (38–40 °C) (Figure 2a). Similarly, pigs did not display any localised or systemic clinical signs (Figure 2b). PRRSV-1 Olot/91 viraemia was inferred by RT-qPCR (Figure 2c). Viral RNA was detected in serum from 3 DPI and remained detectable until the termination of the study (35 DPI). A 2-way ANOVA revealed a significant RNAemia in all three groups with levels not differing significantly between vaccine formulations/routes.

**Figure 3:**
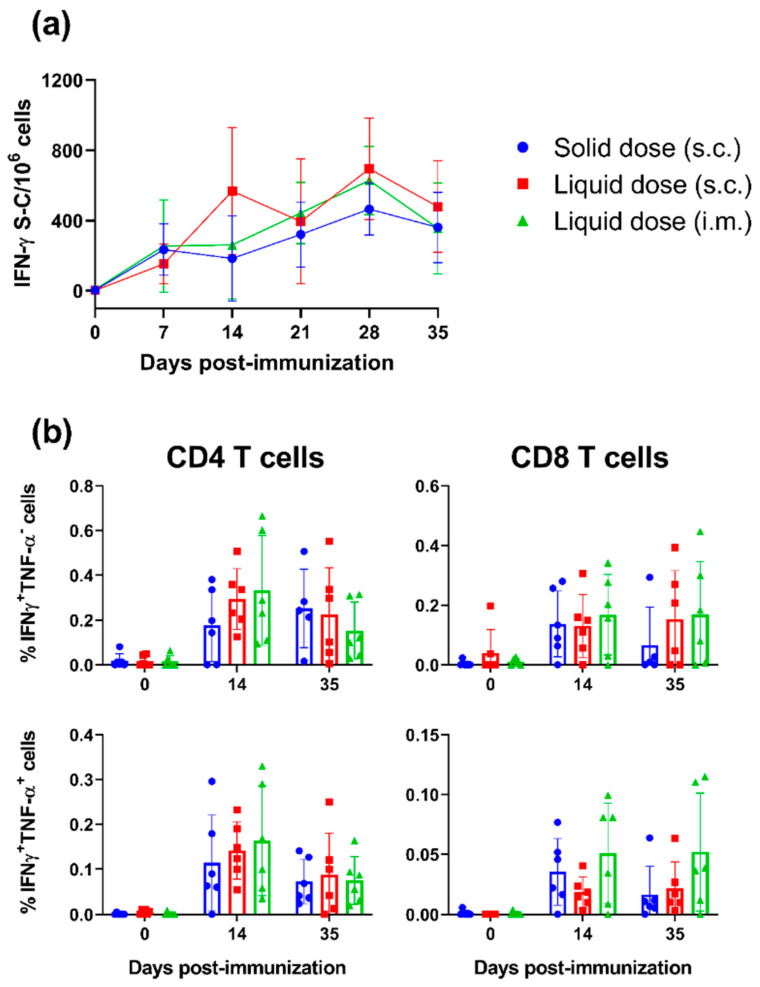
Assessment of T cell responses following immunisation of pigs with a unit solid dose injectable formulation of live attenuated PRRSV-1. Three groups of pigs (n = 6) were immunised by s.c. inoculation of a USDI formulation of 10^5^ TCID_50_ of PRRSV-1 Olot/91, or by s.c. or i.m. inoculation of a comparable dose of PRRSV-1 Olot/91 (s.c. liquid dose and i.m. liquid dose, respectively). Responses of PBMC to stimulation with PRRSV-1 Olot/91 were monitored weekly by (**a**) IFN-γ ELISpot and (**b**) responding cells phenotyped by intracellular cytokine staining (ICS) assays. ELISpot data are presented as the mock-corrected number of IFN-γ spot forming cells (S-C) per million PBMCs, and ICS data shown as the mock-corrected % IFN-γ+TNF-α- and % TNF-α+IFN-γ + CD3 + CD4 + CD8α + (CD4) T cells and CD3+CD4-CD8α+ (CD8) T cells. Mean data ± SD are shown for each vaccine group.

ELISpot assays were performed on longitudinally collected PBMC to enumerate the number of T cells secreting IFN-γ in response to in vitro restimulation with PRRSV-1 Olot/91 (Figure 3a). Significant IFN-γ responses were measurable at all time-points post-immunisation (*p* < 0.001). Responses increased over time with responses on 28 DPI being significantly greater than 7 and 14 DPI. There was no significant difference in the number of IFN-γ spot-forming cells between the groups (*p* = 0.4859). Tukey’s multiple-comparison post-hoc tests revealed that only on day 14 was the response of pigs immunised with the s.c. liquid dose of PRRSV-1 greater than the animals immunised with the solid dose (*p* < 0.05). Intracellular cytokine staining (ICS) assays were performed longitudinally to phenotype T cells responding to PRRSV-1 Olot/91. A standard gating strategy was used to phenotype porcine T cells i.e., CD8 T cells = CD3^+^CD8α^high^CD4^−^; antigen-experienced CD4 T cells = CD3^+^CD8α^low^CD4^+^; naïve CD4 T cells = CD3^+^CD8α^-^CD4^+^; and non-conventional T cells = CD3^+^CD8α^low/-^CD4^-^ [23]. Significant PRRSV-specific cytokine responses were only detected in CD8 and antigen-experienced CD4 T cells (Figure 3b). Across all the vaccine formulations/routes, antigen-experienced CD4 and CD8 T cells expressing IFN-γ and TNF-α and those only expressing IFN-γ increased significantly post-vaccination (*p* < 0.01).

**Figure 4:**
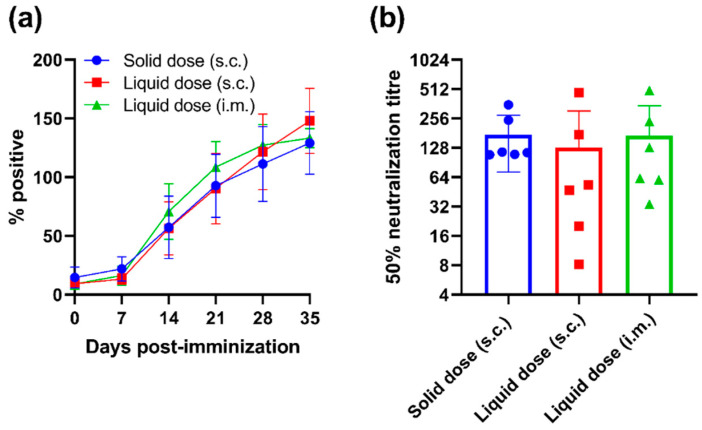
Assessment of antibody responses following immunisation of pigs with a unit solid dose injectable formulation of live attenuated PRRSV-1. Three groups of pigs (n = 6) were immunised by s.c. inoculation of a USDI formulation of 10^5^ TCID_50_ of PRRSV-1 Olot/91, or by s.c. or i.m. inoculation of a comparable dose of PRRSV-1 Olot/91 (s.c. liquid dose and i.m. liquid dose, respectively). (**a**) Longitudinal serum samples were assessed for PRRSV N protein-specific antibodies by ELISA and data presented as percent positive relative to the corrected OD 450 nm max. Results obtained above 30% positive are considered positive. (**b**) PRRSV-1 neutralising antibody titres were assessed in sera from 35 DPI using a virus neutralisation test. Data are presented as the reciprocal serum dilution that neutralised infection by 50% (50% neutralisation titre). Mean data ± SD are shown for each vaccine group.

PRRSV-1 N protein-specific antibodies were detected in the serum from vaccinated pigs by ELISA (Figure 4a). Significant increases in N-protein specific antibody levels were detected in each group from 14 DPI (*p* < 0.01). No significant differences in antibody levels between groups were observed at any timepoint. A virus neutralisation test was conducted using 35 DPI sera to determine PRRSV-1 neutralising antibody titres (Figure 4b). Neutralising antibodies were measurable in the sera of all pigs and there was no significant difference in titres between the vaccine formulations/routes.

### 3.3. Analysis of the Stability of a Solid Dose Vaccine Formulation of PRRSV MLV

**Figure 5:**
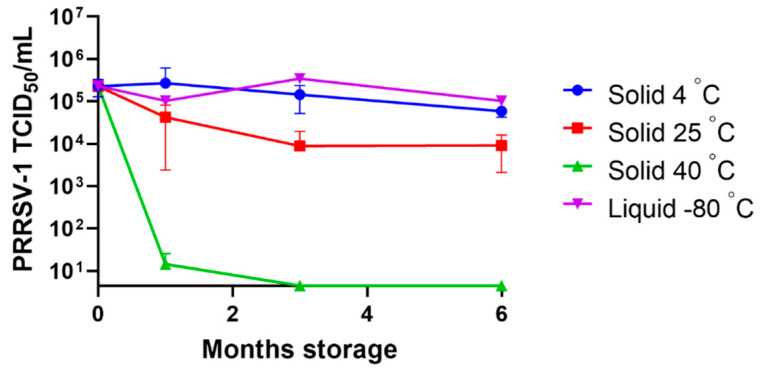
Assessment of the stability of a unit solid dose injectable vaccine formulation of PRRSV MLV. A USDI formulation of PRRSV-1 Olot/91 was stored at 4 °C, 25 °C or 40 °C for 1, 3, and 6 months. At these timepoints, solid doses were reconstituted in DMEM, adjusted to a predicted titre of 1 × 10^5^ TCID_50_/mL and titrated on MARC-145 cells. Untreated PRRSV-1 stored at −80 °C (liquid −80 °C) served as a positive control. Mean data are presented for duplicate or triplicate samples ± SD.

To assess the stability of the USDI vaccine formulation of PRRSV-1 Olot/91, solid doses were stored at 2–8 °C (4 °C), 25 °C or 40 °C for a period of 1, 3, and 6 months. Solid doses were then reconstituted, and infectious titres determined in vitro (Figure 5). After 1-month, solid doses stored at 4 °C and 25 °C retained titre that did not differ significantly from the liquid control virus stored at −80 °C. However, solid doses stored at 40 °C showed a significant drop in titre compared to both the control virus and solid doses stored at the lower temperatures (*p* < 0.001). After 3 months, solid doses stored at 4 °C still retained a titre that did not differ from the control virus, but solid doses stored at 25 °C showed a significant drop in titre (*p* < 0.05). After 6 months, samples stored at 2–8 °C retained a titre of 10^5^ TCID_50_ whereas those stored at 25 °C exhibited a 1.5 log_10_ loss of titre.

## 4. Discussion

This study aimed to develop a novel USDI formulation of live attenuated PRRSV-1 and to evaluate its immunogenicity compared to virus delivered conventionally in liquid suspension by needle-syringe inoculation. Solid dose vaccines are a promising alternative to traditional vaccination methods with several potential advantages such as being needle-free, providing error free and facile dosing, eliminating the need to reconstitute prior to use, and circumventing the cold-chain. In addition, unitary needle-free dosing can improve the safety of the vaccination. This is the first time the USDI technology has been employed for the formulation and delivery of a live attenuated vaccine and the first trial in a large animal model/target species.

An important consideration for USDI vaccine formulation is the freeze-drying and different steps of the manufacturing process which could potentially damage the stability and potency of the vaccine. Therefore, a range of freeze-drying matrix formulations and solid dose manufacturing processes were investigated. All freeze-dried powders and two of three manufacturing processes retained infectious titres of PRRSV as assessed in vitro following reconstitution. The PRRSV vaccine formulation selected based on the highest retention of potency during freeze drying was found to be better suited for the micro-tableting process rather than the cold extrusion (process 1), which is not unexpected. The lead formulation appears inadequate to protect the live PRRSV vaccine during the higher stress process of cold extrusion. The level of retention of potency in solid doses made using process 2 and 3 gave confidence to proceed to evaluate the immunogenicity of a USDI vaccine in pigs. Process 3 was used to manufacture the solid doses as it provided the greatest compressive strength which supports the future development of PRRSV vaccine delivery using the multi-use actuator. High compressive strength is required for the penetration of the skin by the USDI using the multi-use actuator. In this proof-of-principal study, USDI vaccines were delivered using a needle trocar rather than the actuator device. Future studies will evaluate the immunogenicity of USDI vaccines delivered using the actuator. 

The immunogenicity of the USDI formulation delivered subcutaneously was evaluated in comparison with intramuscular or subcutaneous immunisation against a liquid suspension of virus, from the same virus batch. The immunogenicity of a vaccine can depend on individual vaccines and can be influenced by the gender of a vaccinee and the type of adjuvant used [24]. In the current study, the solid dose vaccine induced CD4 and CD8 T cell responses of comparable magnitude and kinetics as the liquid dose. This is significant since cell-mediated immune responses are considered a key component of PRRSV immunity [25]. Neutralising antibody responses are considered another key immune response for protection against PRRSV, with passive immunisation studies having provided direct evidence that these antibodies provide immunity [26]. A virus neutralisation test using the homologous PRRSV-1 Olot/91 was used to assess neutralising antibody titres. Analysis showed comparable responses between solid and liquid formulations There was variation in neutralising antibody titres between pigs, which is commonly seen following PRRSV immunisation/infection, but responses were most consistent in pigs immunised with the solid dose indicating additional advantage of this route of delivery. From these data, it can be concluded that solid dose PRRSV-1 Olot/91 vaccination can induce immune responses that are like those induced by subcutaneous and intramuscular liquid PRRSV-1 Olot/91 vaccines while reducing dose to dose variability and minimising vaccine wastage. The study also demonstrated that subcutaneous and intramuscular routes administered liquid PRRSV-1 Olot/91 vaccines elicited comparable responses. The RT-qPCR data showed comparable levels and kinetics of PRRSV-1 in blood, suggesting that the method or route of inoculation did not have a significant impact on the subsequent course of infection and the concomitant immune responses.

Commercially available lyophilised PRRS MLV vaccines such as MSD Animal Health’s Porcilis® PRRS lyophilizate contain 10^4^–10^6.3^ TCID_50_ per dose with the shelf life of up to two years if stored at 2–8 °C, allowing a generous loss in titre of around 2.0 log_10_ before vaccine efficacy is impacted [27]. Exposure of vaccine to temperatures outside this range during storage or after reconstitution however is contraindicated. PRRSV vaccine formulated as USDI did not lose any infectivity during the study period of six months at 2–8 °C. Additionally, the retention of infectivity of the USDI vaccine at 25 °C meets the current specifications for a PRRS vaccine and, in addition, losses after reconstitution is avoided because the USDI strategy does not require mix-and-shoot administration. 

The recent launch of needle-free vaccine delivery systems onto the animal health market highlights the interest in moving to technologies that bring practical benefits to producers and improve animal health and welfare [28,29], reducing the risk of iatrogenic transmission of pathogens, inadvertent needle stick injuries, and improving meat quality by eliminating needle-induced injection site lesions. This study has demonstrated the potential of the USDI vaccine delivery system as a novel and attractive alternative to conventional delivery of live attenuated PRRSV vaccines.

## Figures and Tables

**Table 1:** List of excipients used in five FDM formulations (✓-presence, X-absence).

FDM Constituents	Formulation				
	1	2	3	4	5
Vaccine	✓	✓	✓	✓	✓
Medium	✓	✓	✓	✓	✓
Gelatin	✓	✓	✓	✓	✓
Albumin	✓	✓	✓	✓	✓
Sorbitol	✓	✓	✓	✓	✓
Mannitol	✓	✓	✓	✓	✓
Sucrose	✓	✓	✓	✓	✓
Trehalose	✓	✓	✓	X	X
PEG	✓	X	X	X	X
PVP	X	✓	X	X	✓

## Data Availability

Not applicable.
